# Hospital Depression and the Registration of Nurses

**Published:** 1887-10-22

**Authors:** Andrew Clark

**Affiliations:** President of The Hospitals Association


### Hospital Depression and the Registration of Nurses.

By Sir Andrew Clark, Bart., M.D., F.R.S.,
President of The Hospitals Association.

I suppose I need scarcely tell you that the primary object of The Hospitals Association is to help in the great work of the hospitals both in this great city and throughout the provinces; to help them to make their work more economical and more efficient, if that may be. Now, most of you know that the contributions for the support of these great hospitals, at all events the great hospitals of London, have been steadily diminishing, and I can contemplate no disaster greater for medical knowledge, or for society which benefits by medical knowledge, than the dwindling away, to any great extent, of the contributions made for the support of the hospitals. You know that they not only help to cure the sick, but that they also discharge a duty which is only second in importance to it—the duty of originating, expanding, correcting, and confirming knowledge—a duty which every one in this room benefits by to a greater or less extent. Now, what is the reason that the support to our great hospitals is diminishing? I am not going to discuss the theories which have been propounded, but I believe there is one explanation which in truth and importance transcends all others. It is this: I have such a firm belief in the philanthropy of Englishwomen that I feel satisfied, nay, I feel absolutely convinced, that if they knew really and completely the work done by the hospitals, the contributions to them would be more than are required. But they do not know it; they do not know, even in London, the work which is being done, far less do they know it in the provinces. Now this Hospitals Association wishes not only to help the hospitals in their work, but to fill this very function, to assist in making known, not only to the people of London but to the people in the provinces, the need, extent, and importance of this work which is being done, and in order to make this help as large, as efficient, as wide as it can be made, the Association proposes—indeed, it has already proposed—to form local associations throughout the length and breadth of the land which shall be complete in themselves, each complete in itself, each having an autonomy, but each connected with the others, and all affiliated with the central institution in London. Another subject in which The Hospitals Asssociation is particularly interested is in the registration of nurses. We do not only mean to help hospitals, but also to help those who are connected with them; and there is, I believe no class of persons connected with hospital work more important than the nursing class. We all feel that we owe nurses a great deal, and in making this acknowledgment we are anxious that their reputation for character, for competence, and for loyalty to their duties shall be placed beyond dispute. Now this cannot be said at the present moment. I do not wish to cast any reflection upon so great and important a body, but, taken as a class, it cannot now be said that they are all of them efficient, that they are all of them always exactly the sort of characters we should wish for the sake of the body itself. Now, we should like to have every nurse registered, to affiliate her with one of these associations, to have her affiliated upon certain grounds which would render her character and competence unquestionable. This we propose to do roughly in this way—first, we require that every nurse shall have passed through a certain training; secondly, that she shall have had a qualifying certificate for that training; and thirdly, that she shall have discharged a certain portion of a nurse's duty for a certain period with competence and loyalty to her duty, and that she shall have a certificate to that effect. Now, if we carry out this registration in the way we desire to carry it out, it will not only raise officially the standard of nurses, but it will be of inexpressible good to families employing them. Many of the nurses sent into private families are sent from institutions of various kinds. They are commercial institutions, which have not the power of ascertaining what is their exact character, their exact competence, and whether they have discharged their duty as they should. This power, then, we should like to have for the sake alike of the nurses and of the society which employs them. If all who read this would only help us to carry out this purpose, I am sure you would be doing a real and a great work, not only important to nurses as a body, but to society at large.

*** Dr. Steele, Superintendent of Guy's Hospital, will read a paper on the Registration of Nurses, at the Society of Arts, John Street, Adelphi, W.C., at 8 p.m. on Wednesday, December 14th next.

